# Will metformin use lead to a decreased risk of thyroid cancer? A systematic review and meta-analyses

**DOI:** 10.1186/s40001-023-01287-0

**Published:** 2023-09-29

**Authors:** Hailong Li, Yue Chen, Lei Hu, Wenzhi Yang, Zongshi Gao, Mengqing Liu, Hui Tao, Jie Li

**Affiliations:** 1 Department of Clinical Medicine, Sun Yat-Sen University, No.74 Nonglin Road, Guangzhou, 510030 Guangdong People’s Republic of China; 2 Department of Orthopedics, The First Affiliated Hospital of Anhui Medical University, Hefei, 230022 Anhui People’s Republic of China; 3 Department of Spine Surgery, The First Affiliated Hospital of Anhui Medical University, Hefei, 230022 Anhui People’s Republic of China; 4 Department of Clinical Medicine, School of Chaohu Clinical Medicine, Anhui Medical University, No. 81 Meishan Road, Hefei, 230032 Anhui People’s Republic of China; 5 Department of Clinical Laboratory, The First Affiliated Hospital of Anhui Medical University, Hefei, 230022 Anhui People’s Republic of China

**Keywords:** Metformin use, Thyroid cancer, Systematic review, Meta-analyses

## Abstract

**Background:**

It has been reported that metformin use may reduce the risk of thyroid cancer, but existing studies have generated inconsistent results. The purpose of this study was to investigate such association between metformin use and the risk of thyroid cancer.

**Methods:**

Studies of metformin use for the risk of thyroid cancer were searched in Web of Science, PubMed, Embase, Cochrane Library, China National Knowledge Infrastructure, China Biomedical Database, Wanfang Data, and Chinese Scientific Journals Database (VIP) from the establishment date to December 2022. Newcastle–Ottawa scale is adopted for assessing the methodological quality of included studies, and the inter-study heterogeneity was assessed by using the *I*-squared statistic. Combined odds ratios (ORs) with the corresponding 95% confidence intervals (CIs) were calculated through either fixed-effects or random-effects model according to the heterogeneity. Besides, subgroup analyses, sensitivity analyses and test for publication bias were conducted.

**Results:**

Five studies involving 1,713,528 participants were enrolled in the qualitative and quantitative synthesis. The result of the meta-analyses showed that metformin use was associated with a statistically significant lower risk of thyroid cancer (pooled OR = 0.68, 95% CI = 0.50–0.91, *P* = 0.011). Moreover, in the subgroup analysis, we found that the use of metformin may also aid in the prevention of thyroid cancer in Eastern population (pooled OR = 0.55, 95% CI = 0.35–0.88, *P* = 0.012) rather than Western population (pooled OR = 0.89, 95% CI = 0.52–1.54, *P* = 0.685). Sensitivity analysis suggested the results of this meta-analyses were relatively stable. No publication bias was detected.

**Conclusion:**

Metformin use is beneficial for reducing the risk of thyroid cancer. For further investigation, more well-designed studies are still needed to elucidate the association between metformin use and the risk of thyroid cancer.

## Introduction

Thyroid cancer is a common malignant tumor of the endocrine system, and its incidence is increasing worldwide [1]. According to the GLOBOCAN 2020 estimation, there were 586,000 cases of thyroid cancer globally, accounting for 3.0% of the total incidence of malignancies [2]. Thyroid cancer can be classified into papillary thyroid cancer (PTC), follicular thyroid cancer (FTC), medullary carcinoma and undifferentiated carcinoma. PTC and FTC are collectively known as differentiated thyroid cancer, which is characterized by slow progression, low mortality rate and good prognosis [3]. Nevertheless, considering the gradual increase in the number of thyroid cancer cases, exploration of protective factors that are associated with the prevention of thyroid cancer is crucial for the management of thyroid cancer. Metformin is currently one of the most widely used oral hypoglycemic agents in the world, and is also a core drug in the prevention and control of diabetes worldwide [4]. As the use of metformin becomes more widespread, numerous studies showed that metformin might also played essential roles in protecting the cardiovascular system, slowing down the aging process, and inhibiting tumor progression [5–7]. Moreover, many clinical trials have reported its positive effects on liver, lung, gastric, esophageal, and thyroid cancer [8–12]. In recent years, many studies have shown that metformin can exert anti-division and pro-apoptotic effects in thyroid cancer cell lines and enhance the anti-proliferative effects of chemotherapeutic drugs such as doxorubicin and cisplatin. Metformin can inhibit insulin-induced growth stimulation in differentiated and undifferentiated thyroid cancer and thyroid cancer stem cells, and these effects of metformin are closely related to insulin/IGF signaling and AMPK/mTOR pathways [13]. The conclusion found by Han et al. that metformin may inhibit the growth, migration and epithelial–mesenchymal transition (EMT) of thyroid cancer cell lines through mTOR pathway other than insulin pathway is also consistent with the above findings [14]. In vitro studies have shown that metformin inhibits the growth of primary thyroid cells and thyroid cancer cells by reducing hyperinsulinemia and direct cell effects, including inhibiting cell cycle progression and inducing apoptosis [15]. In animal models, metformin can inhibit the progression of obesity-activated thyroid cancer [16, 64]. A recent study has shown that the tumor size of thyroid cancer patients treated with metformin is small, indicating that the drug has an inhibitory effect on tumor growth. Vitro data showed that p70S6K/pS6 signal transduction may be a molecular target of metformin in thyroid cancer cells [17]. Moreover, a large observational study of T2DM patients in Taiwan found that metformin reduced the risk of thyroid cancer [18]. In addition, population-based observational studies suggested reduced tumor incidence and cancer-related mortality in patients with long-term metformin use in esophageal cancer, hepatocellular cancer, colon cancer, etc. [19–21]. All of this suggests that research into the role of metformin in thyroid cancer continues, with studies of metformin's role in the development and prognosis of thyroid cancer becoming clearer. However, in thyroid cancer, it is not clear whether metformin can reduce the incidence of cancer. Cho et al. found that metformin appeared to be associated with a preventive effect on thyroid cancer development in a nationwide population-based study [22]. However, the outcome of Becker’s study concluded that the use of metformin was not associated with a decreased risk of thyroid cancer [23]. The findings of a previous meta-analysis by Wang et al. also did not support that metformin use was associated with decreased risk of thyroid cancer [24]. However, other studies have been published since the publication of the meta-analysis with contrasting results. Thus, this updated meta-analyses was performed to investigate whether metformin had a protective effect on the prevention of thyroid cancer.

## Methods

This study was conducted with the requirements of Preferred Reporting Items for Systematic Review and Meta-Analyses (PRISMA) guideline [25].

### Search strategy

A comprehensive search of original studies investigating the association between metformin use and thyroid cancer was carried out in three English databases (Web of Science, PubMed, and Cochrane Library) and four Chinese databases (China National Knowledge Infrastructure, China Biomedical Database, Wanfang Data, and VIP database) to obtain eligible articles from the inceptions of databases to January 2023. We used the following searching terms: (metformin OR dimethylguanylguanidine) AND (thyroid) AND (adenocarcinoma OR carcinoma OR malignancy OR tumor OR neoplasm OR squamous carcinoma OR cancer). In addition, the reference lists of the included articles and conference records were also reviewed to identify other potentially inclusive studies.

### Inclusion and exclusion criteria

Studies for inclusion must meet the following criteria: (1) the study design should be cohort studies or case–control studies. (2) Metformin use was the intervention or exposure for the exposure group and any feasible intervention without metformin use can be implemented on the control group. (3) The ending indicator was the incidence of thyroid cancer. (4) The study reported relative risks (RRs), hazard ratios (HRs) or odd ratios (ORs) with the corresponding 95% confidence intervals (CIs) or providing sufficient data for calculating the effect size between metformin use and thyroid cancer.

Studies with the following characteristics were excluded: (1) the type of articles were not reported or could not be determined. (2) The type of literature was review, in vivo studies, in vitro studies or case reports. (3) The effect size could not be extracted or recalculated. (3) Duplicate studies were also excluded.

### Data collection and quality assessment

To ensure the accuracy and objectivity of the data, two evaluators (Hailong Li and Yue Chen) independently screened the literature, extracted information, and evaluated the quality of the studies. These processes need to be cross-checked, and if there was disagreement between the two authors, a third person (Lei Hu) was consulted to assist in the judgment.

The articles were screened by first reading the title, and after eliminating obviously irrelevant literature, the abstract and full text were further read to determine the final inclusion. A pre-defined data collection form was used to collected the information. Data extracted from studies included: the first author’s name, publication year, study design, data source, location, sample size, the age of the participants, study period, assessment method of thyroid cancer, variables adjusted in the statistical analyses, quality assessment score, and effect estimates with the corresponding 95% confidence intervals (CIs).

The Newcastle–Ottawa Quality Assessment Scale (NOS), a tool for quality assessment of non-randomized studies was employed to evaluate the quality of each study [26]. It consisted of eight items classified into three dimensions, including group selection, groups comparability, and exposure/outcome. The maximum scores of this checklist were nine, and scores between seven and nine were rated as high study quality [26].

### Statistical analysis

The STATA statistical software (version 14.0; StataCorp, College Station, TX, USA) was employed to perform the data analyses. Considering the relative low incidence of thyroid cancer, HRs were viewed as ORs, and the results were analyzed through pooling the adjusted ORs with 95% CIs by inverse variance method. Q statistic and *I*^2^ statistic were used to quantitatively assess the extent of heterogeneity of the studies [27]. Values of *P* < 0.10 and *I*^2^ > 50% were considered to be representative of having statistically significant heterogeneity and the random-effects model was then applied for pooling the results. Otherwise, a fixed-effects model was used to create forest plots [28]. Subgroup analyses were performed by classification by study design (case–control studies or cohort studies), geographic locations (Western countries or Eastern countries), the sample size (< 10,000 or ≥ 10,000), and study quality (moderate or high). Sensitivity analyses were conducted by changing the random/fixed-effects model [29–31]. In addition, publication bias was assessable on a funnel plot qualitatively and Begg’s test and Egger’s test quantitatively. P values less than 0.05 was considered statistically significant [32, 33].

## Results

### Study selection and study characteristics

**Fig. 1:**
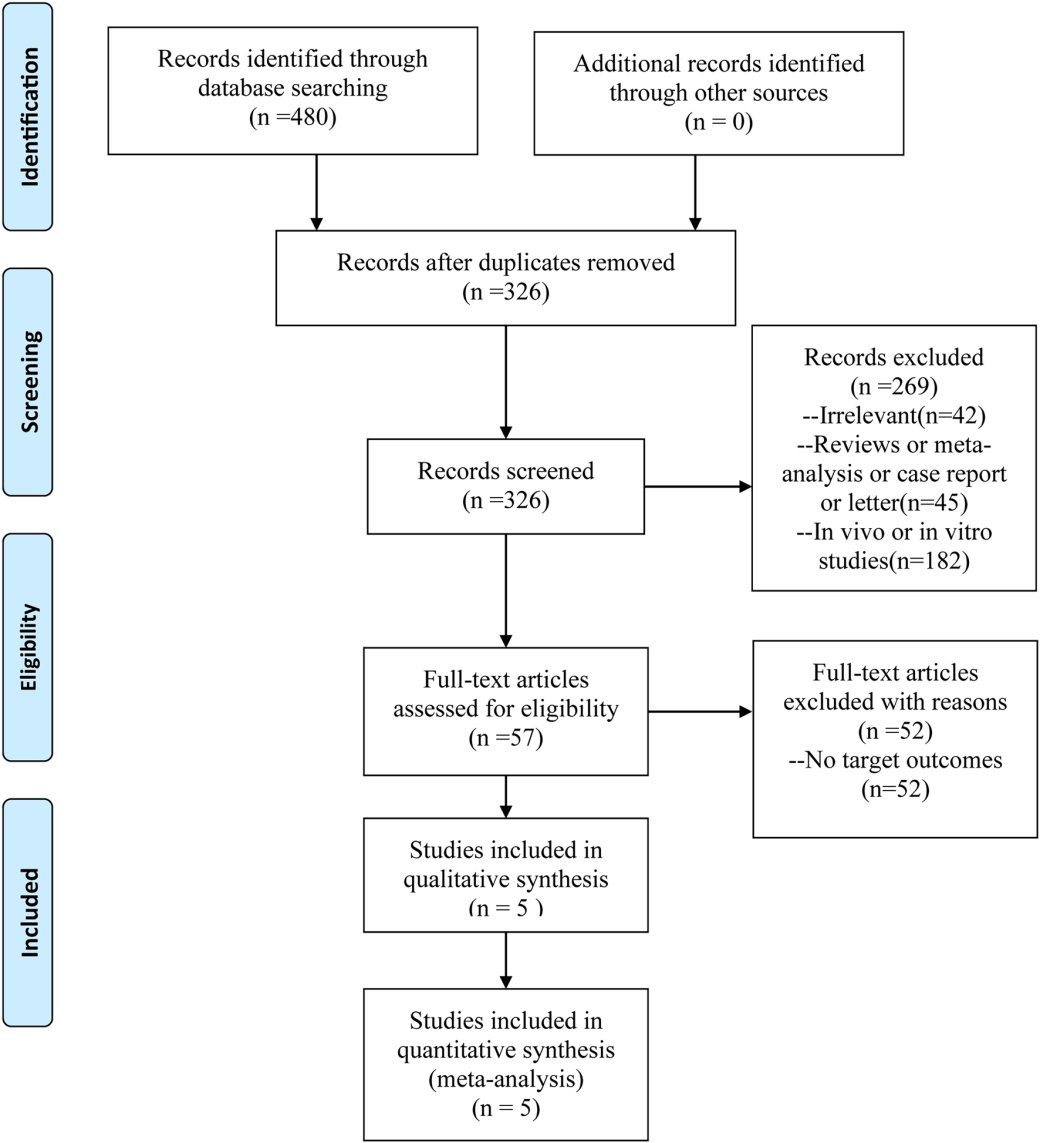
PRISMA flowchart

A total of 480 relevant articles were identified based on the searching terms presented in the method section by initial search across all databases searched. No additional studies were identified through other sources. A total of 154 duplicate articles were removed, and 269 articles were excluded by screening the titles or abstract. During the second stage of full-text screening, we further excluded 52 studies, resulting in five full-text articles [18, 22, 23, 34, 35] for the subsequent data extraction and synthesis. The outline of the inclusion process is presented in Fig. 1.

In this meta-analyses, we identified five studies [18, 22, 23, 34, 35] with 1,713,528 participants, published between 2014 and 2022, which included three cohort studies and two case–control studies. Three studies were conducted in Western countries, two in Eastern countries. The population sizes of the included studies ranged from 291 to 1,414,723. The included studies were all of high or moderate quality. Detailed characteristics of included studies are summarized in Table 1.

**Table 1:** Characteristics of studies included in this meta-analysis

Study	Study design	Data source	Location	Sample size	Age	Study period	Assessment method of thyroid cancer	Estimate effect (95% CI)	NOS scores	Adjusted confounders
Becker 2015	Case–control	UK-based Clinical Practice Research Datalink	United Kingdom	8603	Not specified	From January 1995 to December 2014	NA	OR:1.42 (0.74–2.73)	7	Adjusted^a^
Cho 2017	Cohort	Korean National Health Insurance claim database	Korea	256,906	Aged ≥ 30 years	From January 2005 to December 2009	ICD-10	HR:0.69 (0.60–0.79)	7	Adjusted^b^
Luo 2016	Cohort	The Women’s Health Initiative in USA	United States	33,005	50–79 years old	From September 1993 to December 1998	Self-administered questionnaires	HR:0.99 (0.46–2.12)	6	Adjusted^c^
Sulu 2022	Case–control	Database of Patients with Acromegaly in the own university hospital	Turkey	291	Above 18 years of age	From January 1983 to December 2019	Medical READ codes	OR:0.62 (0.67–0.83)	8	Adjusted^d^
Tseng 2014	Cohort	National Health Insurance database	China	1,414,723	Not specified	From 1996 (the earliest database available) to 2009	ICD-9-CM	HR:0.43 (0.34–0.56)	8	Adjusted^e^

^a^Adjusted for other antidiabetic drugs in the table, BMI, smoking, diabetes mellitus, hyperthyroidism, and goiter

^b^Adjusted for age, sex, year of study enrollment, income, living area, and antidiabetic drugs other than metformin

^c^Adjusted for age at enrollment, ethnicity, education, smoking status, recreational physical activity, alcohol intake, history of HT use, and previous thyroid disease

^d^All variables are scaled to prevent range related bias in conditional logistic regression

^e^Adjusted for age, the severity/duration of diabetes, other antidiabetic drugs used, hypertension, gender, other cancer, chronic diseases status, medication

### Overall meta-analyses

**Fig. 2:**
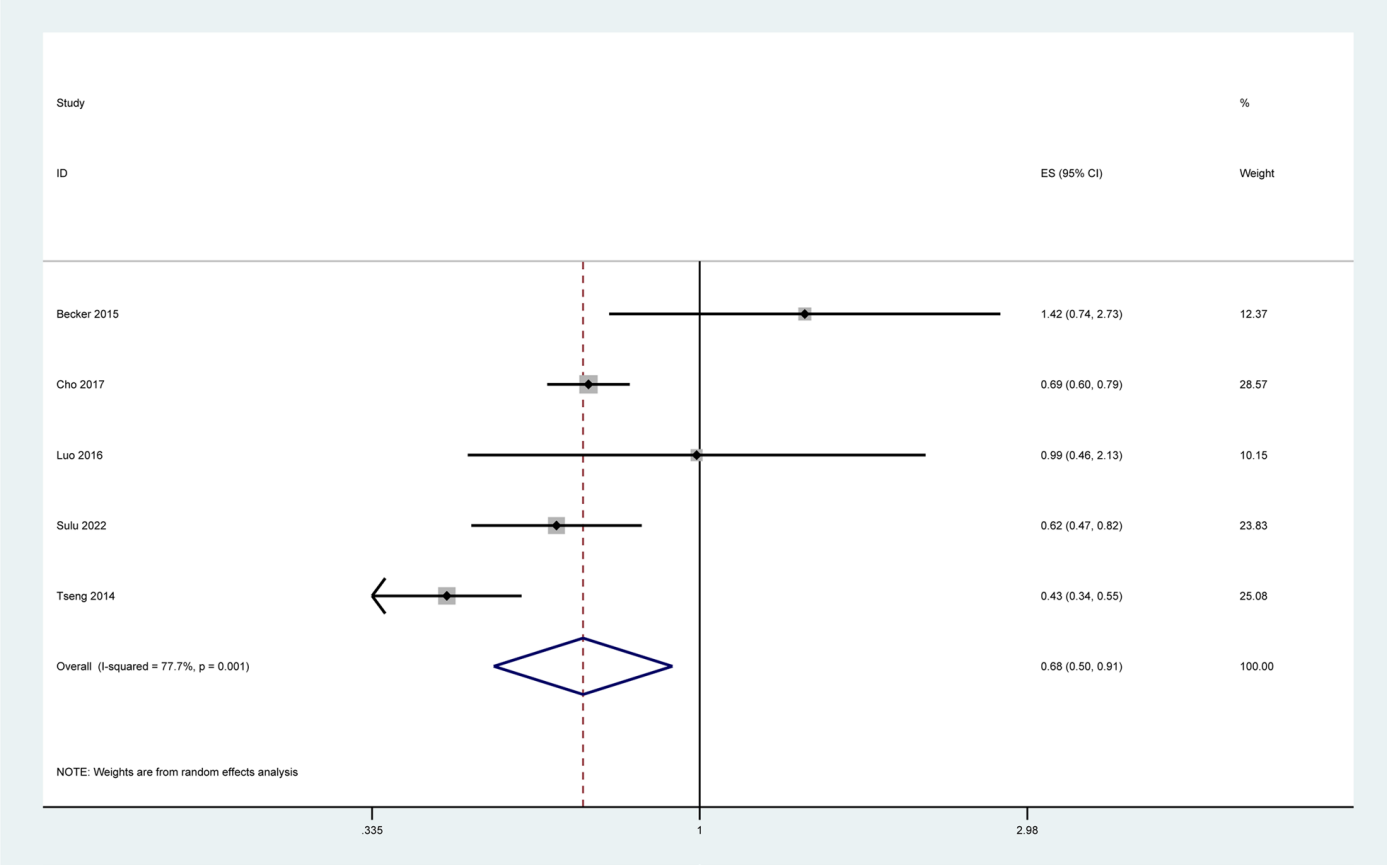
Forrest plot: association between metformin use and the risk of thyroid cancer

Five articles [18, 22, 23, 34, 35] regarding the association between metformin use and thyroid cancer were included for meta-analyses. The forest plot displayed the pooled OR under the random-effects model. The pooled result indicated that metformin use was associated with reduced risk of dementia (pooled OR = 0.68, 95% CI = 0.50–0.91, *P* = 0.011; Fig. 2).

### Subgroup analyses

In the subgroup analysis by study design, statistically significant associations were observed in cohort studies (pooled OR = 0.61, 95% CI = 0.41–0.92, *P* = 0.018) while no such relationship was detected in case–control studies (pooled OR = 0.89, 95% CI = 0.40–1.99, *P* = 0.774). When stratified by geographic locations, the results were different when confined to different subgroups (Eastern countries: pooled OR = 0.55, 95% CI = 0.35–0.88, *P* = 0.012; Western countries: OR = 0.89, 95% CI = 0.52–1.54, *P* = 0.685). For sample size, two studies that followed the study with the sample size ≥ 10,000 indicated a significant impact of metformin use on reducing the risk of developing thyroid cancer (pooled OR = 0.61, 95% CI = 0.41–0.92, *P* = 0.018). However, in the two studies with sample size of < 10,000, the association between the metformin use and incidence of thyroid cancer was not statistically significant (pooled OR = 0.89, 95% CI = 0.40–1.99, P = 0.774). Further analysis of four high-quality studies regarding its effect on thyroid cancer also find a statistically significant protective effect (pooled RR = 0.65, 95% CI = 0.47–0.89, *P* = 0.008). There was only one study of moderate quality, which reported no significant reduction in thyroid cancer (OR = 0.99, 95% CI = 0.46–1.12, *P* = 0.979). All these results of the subgroup analyses are represented in Table 2.

**Table 2:** Summary of pooled ORs with CI in the meta-analysis

Group	No. of studies	Heterogeneity		Significant		OR (95%CI)	Model
		*I*^2^ (%)	*P*_heterogeneity_	*Z*	*P*		
Overall	5	77.7	0.001	2.56	0.011	0.68 (0.50–0.91)	Random
Study design							
Case–control studies	2	80.8	0.023	0.29	0.774	0.89 (0.40–1.99)	Random
Cohort studies	3	83.3	0.002	2.36	0.018	0.61 (0.41–0.92)	Random
Geographic locations							
Western	3	65.9	0.053	0.41	0.685	0.89 (0.52–1.54)	Random
Eastern	2	90.6	0.001	2.52	0.012	0.55 (0.35–0.88)	Random
Sample size							
< 10,000	2	80.8	0.023	0.29	0.774	0.89 (0.40–1.99)	Random
≥ 10,000	3	83.3	0.002	2.36	0.018	0.61 (0.41–0.92)	Random
Quality							
Moderate	1	NA	NA	0.03	0.979	0.99 (0.46–2.12)	NA
High	4	82.0	0.001	2.65	0.008	0.65 (0.47–0.89)	Random

OR: odds ratio; CI: confidence Interval; NA: not applicable

### Sensitivity analyses and bias diagnostics

**Fig. 3:**
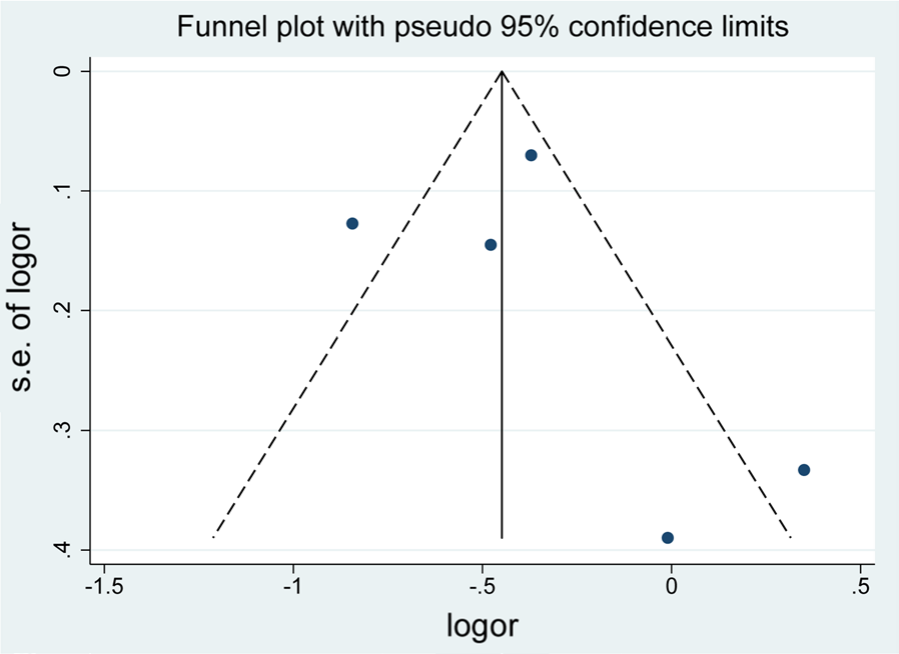
Funnel plot

We conducted a sensitivity analysis by changing the random/fixed-effects model, which suggested the statistical stability of this meta-analyses. No publication bias was detected in the analysis of the association between metformin use and dementia/Alzheimer’s disease (Begg's test: *Z* = 0.24, *P* = 0.806; Egger's test: = 0.48, *P* = 0.664. Symmetrical funnel plot is shown in Fig. 3).

## Discussion

Meta-analyses are considered to be an important tool for more accurately assessing the risk of disease development and the effectiveness of treatment [36, 37]. Based on the fact that metformin has significant clinical therapeutic and preventive effects on many cancers, including colorectal [38] and lung cancers [39], and that several studies have reported on the potential use of metformin in the treatment of thyroid cancer [15], but no definitive study has yet shown that metformin reduces the risk of thyroid cancer, we conducted this updated meta-analyses. The results found that patients taking metformin had a 0.68-fold reduced risk of developing thyroid cancer. This differs from the results of a previously relevant meta-analyses, which found no statistically significant association between metformin use and the risk of developing thyroid cancer, most likely due to the inclusion of a larger number of studies in this meta-analyses. The funnel plot analysis showed no significant publication bias, which also supports the stability of the study's results.

Subgroup analysis showed that metformin use was associated with a 45% reduction in thyroid cancer incidence in Eastern countries, compared with only an 11% reduction in Western countries, and this result was not statistically significant in Western countries. Firstly, there is a statistically significant association between metformin and a significant reduction in the incidence of thyroid cancer in Eastern countries compared to Western countries. Thyroid cancer is influenced by a number of factors, including overexpression, mutation or deletion of proto-oncogene sequences [40], iodine intake (iodine deficiency or high iodine) [41], gender and age, and alcohol consumption [42] and stress are all predisposing factors. Among the Eastern countries, China and Korea are the two major countries with a high prevalence of thyroid cancer, and thyroid nodules are quite common in the general population in these regions, with their incidence increasing with the age of the patient [43]. Thyroid nodules are closely associated with thyroid cancer, with some thyroid cancer coming from thyroid nodules [44]. Independent factors associated with an increased risk of thyroid nodules include being female, having diabetes, high blood pressure, high betel nut and red meat consumption, while underweight appears to be protective [45]. It has been shown that women with previous diabetes have an increased risk of developing thyroid cancer compared to non-diabetic patients [46]. According to the latest data from the International Diabetes Federation (IDF), the high prevalence of diabetes in Eastern countries, as represented by China [47], may be one of the reasons why metformin significantly reduces the incidence of thyroid cancer in Eastern countries. However, in Western countries, overdiagnosis, radiation exposure, iodine deficiency and volcanic activity are all important factors contributing to the increased incidence of thyroid cancer in the population [43], changes in these factors were not significantly associated with metformin, which may explain the small reduction in thyroid cancer incidence in Western countries and the lack of significance of metformin in the analysis. However, we did not perform further subgroup analyses in this regard due to lack of raw data. Essential differences in study design may explain the differences in the results of the cohort and case–control studies. Recall and selection bias are inherent in case–control studies, and the order of exposure and disease is equally difficult to determine.

**Fig. 4:**
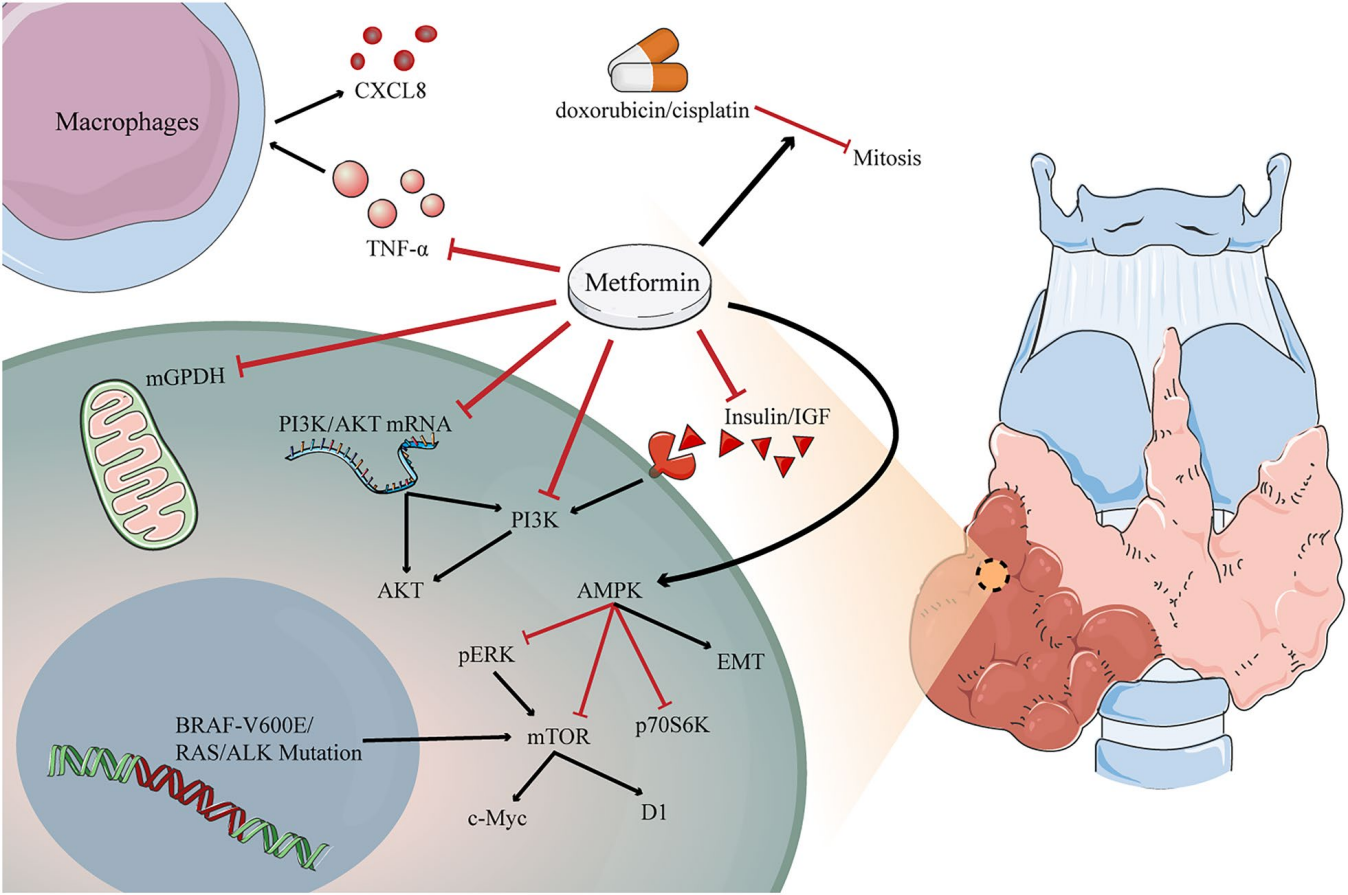
Potential mechanisms of metformin in the prevention and treatment of thyroid cancer

There is a growing body of literature on the beneficial effects of metformin in the prevention and management of cancer (Fig. 4). In addition to its antidiabetic effects, metformin can also inhibit tumor growth in various cancers [48–50]. Metformin can act on various cellular mechanisms in the development of cancers associated with diabetes and obesity through its anti-inflammatory and anti-oxidative stress functions [38]. Metformin has two major potential anticancer mechanisms mediated through interference with insulin/IGF signaling and the AMPK/mTOR pathway, respectively [13]. Metformin can improve insulin sensitivity, thereby inhibiting the insulin-induced mitogenic pathway [51]; it can also can reduce IGF secretion, thereby affecting the body's energy uptake [52]. In addition, metformin can inhibit cell proliferation by reducing IGF levels [53]. AMPK has a considerable role in homeostatic energy biological responses at both the cellular and whole organism level, and metformin can inhibit anabolism and promote catabolism through AMPK activation, thereby altering the body's energy metabolism, while AMPK activation can inhibit DNA production in tumor cells, thereby keeping them in the G1 phase and thus acting as an inhibitor of tumor cell proliferation [51, 54, 55]. In addition, metformin promotes apoptosis in cancer cells by regulating the production of anti-inflammatory and pro-inflammatory mediators [38, 56]. It has been shown that metformin induces anti-inflammatory properties, inhibits DSS-induced IκB kinase activation and reduces colon carcinogenesis in IL-10−/− mice by increasing AMPK activation in intestinal epithelial cells [38]. The target protein of rapamycin (mTOR) is a conserved protein kinase that is commonly found in mammals. mTOR is an important link in the cellular signaling process, and mTOR signaling plays a key role in human cancers [57]. Metformin can inhibit the mTOR pathway by activating AMPK, which may lead to reprogramming of cancer metabolism, which in turn exerts inhibitory effects on cancers such as liver, colorectal and breast cancers [38, 58, 59]. mTOR can be inhibited by proteins involving protein kinase B/AKT and abnormal activation of upstream signaling involving extracellular signal-regulated kinase (ERK) [60]. In addition to these kinases, AMPK can regulate mTOR signaling by promoting activation of the TSC1/2 complex [61]. AMPK can also induce catabolism and downregulate cell proliferation by mimicking a state of caloric deprivation, mechanisms that have been associated with metformin-mediated inhibition of cancer cell growth [51].

The effect of metformin on thyroid cancer has been a hot topic of research in recent years. Metformin inhibits all thyroid cancer cell lines and its effects broadly affect cell proliferation, apoptosis and cell cycle arrest, as well as colony formation and migration [62]. Clinical trials have shown that metformin treatment is associated with higher remission and survival rates in diabetic patients with thyroid cancer [17]. Another large observational study in Taiwanese patients with type 2 diabetes mellitus (T2DM) found that metformin reduced the risk of thyroid cancer [18]. Metformin has been shown to inhibit TNF-α induced CXCL8 secretion in primary cultures of human thyroid cells and thus be indirectly anticancer [63]. Metformin also exerts growth inhibitory effects on primary thyroid cells and thyroid cancer cells by reducing hyperinsulinemia and direct cellular effects, including inhibition of cell cycle progression and induction of apoptosis [13]. In addition, metformin significantly reduces the effect of insulin on differentiated human thyroid cells, mesenchymal thyroid cancer cells, adriamycin-resistant thyroid cancer cell lines and thyroid cancer stem cells by growth stimulation [15]. In a mouse model, metformin blocks the progression of obesity-activated thyroid cancer [64].

mTOR pathway targets are activated in thyroid cancer, and metformin may also inhibit the growth, migration and mesenchymal transition of thyroid cancer cell lines through the mTOR pathway other than the insulin pathway [14]. Mutations in the BRAF-V600E gene occur in approximately 45% of papillary thyroid cancers [65], while mutations in the RAS and ALK genes are frequently found [66–68] These mutations drive mTOR pathway activation and lead to thyroid tumourigenesis [69]. The AMPK pathway is closely connected to the mTOR pathway and metformin can significantly activate the AMPK pathway in a dose-dependent manner, thereby inhibiting the expression of cell cycle protein D1 and c-Myc by downregulating p-mTOR (mTOR phosphorylation). As proto-oncogenes, cyclin D1 and c-Myc play key roles in tumor cell growth, migration and invasion [50, 70–73]. In papillary thyroid carcinoma (PTC) and follicular thyroid carcinoma (FTC)-derived cell lines, metformin inhibited cancer cell growth and down-regulated cyclin D1 expression. In an oxidative stress model, metformin enhanced H2O2-induced activation of AMPK activity and inhibited pERK and p70S6K activity in PTC and FTC-derived cells. In addition, metformin antagonized the growth-stimulating effects of insulin, inhibited clonal cell growth, reduced thyroid cancer spheroid formation and enhanced the antimitotic effects of chemotherapeutic agents (doxorubicin and cisplatin) on anaplastic thyroid cancer (ATC) cells [13, 74]. In medullary thyroid carcinoma (MTC)-derived cells, metformin reduced the expression of cyclin D1 and inhibited cell growth. Metformin treatment was associated with inhibition of mTOR/p6S6K/pS1 signaling and downregulation of pERK in MTC-derived TT and MZ-CRC-70 cell lines [75]. EMT underlies cancer cell invasion and metastasis and is a key event in the progression of many cancers [76]. The malignant progression of many types of cancer depends on EMT activation in tumor cells [77–79]. Early studies have shown that multiple molecules can induce EMT, including transforming growth factor β (TGFβ), epidermal growth factor (EGF), protein kinase B (AKT) and ERK [80, 81]. A study reported that elevated thyroid hormone (TH) levels promote the EMT and malignant evolution of squamous cell carcinoma (SCC) cells. TH induces EMT by transcriptionally up-regulating ZEB-1, mesenchymal genes and metalloproteinases, and inhibits the expression of E-cadherin, which further illustrates the important potential role of EMT in the development of thyroid cancer [82]. And studies have shown that EMT is very common in thyroid cancer invasion [83]. Metformin can inhibit thyroid cancer cell proliferation, migration, invasion and EMT by activating AMPK and subsequently inhibiting mTOR [14].

Both insulin and IGF-1 can induce cell growth by stimulating the PI3K/AKT signaling pathway [84]. Metformin can affect cell proliferation by regulating the PI3K/AKT signaling pathway, and metformin may significantly inhibit the proliferation of ATC cell lines by downregulating the mRNA expression of PI3K and AKT in the PI3K/AKT signaling pathway without affecting PI3K or AKT phosphorylation [84]. Mitochondrial glycerol phosphate dehydrogenase (mGPDH) is an essential component of the mitochondrial respiratory chain and plays a key role in the phosphoglycerol shuttle as a rate-limiting step [85]. mGPDH has been shown to be overexpressed in thyroid cancer which also leads to increased thyroid cancer cell growth and stimulates mitochondrial respiration to meet the metabolic demands of increased proliferation [86]. mGPDH is a metformin cancer targets for cancer and metformin treatment is associated with reduced mGPDH expression, growth inhibition and inhibition of mitochondrial respiration and a shift in cancer cell metabolism towards glycolysis [86, 87]. mGPDH silencing reduces the sensitivity of metformin to inhibit mitochondrial oxidative phosphorylation (OXPHOS) and growth, whereas mGPDH overexpression sensitizes thyroid cancer cells to the growth inhibitory effects of mitochondrial respiration and metformin. mGPDH is a novel thyroid cancer growth and metabolism regulator that can be effectively targeted by metformin [86].

However, the meta-analyses we conducted also had certain limitations. Firstly, there were insufficient primary study data included to allow for a more in-depth subgroup analysis of thyroid cancer by gender, age, other health status and specific environmental factors in the populations investigated in both Eastern and Western countries. Therefore, the specific reasons for the analytical finding that metformin use had no significant effect on reducing the prevalence of thyroid cancer in Western countries remain to be investigated, as well as the specific mechanisms by which metformin reduces the occurrence of thyroid cancer in Eastern countries and its exact causes still need to be explored in more depth. Secondly, as all included studies were observational, we were unable to analyze with certainty the specific reasons for the results. Finally, we found a large heterogeneity. However, we performed meta-regression and subgroup analyses to investigate the impact.

Despite these limitations, our meta-analyses has some significant advantages. Firstly, the assessment of publication bias showed relatively stable results with insignificant publication bias. Secondly, more studies were included this time than in the previous meta-analyses, which resulted in relatively enhanced statistical power, and we performed a more comprehensive subgroup analysis in this study. In addition, we have provided a detailed account of the mechanisms of metformin action in thyroid cancer, illustrating the possibility of metformin inhibition of thyroid carcinogenesis from a mechanistic review, which also provides a better theoretical basis for the meta-analyses.

## Conclusion

In conclusion, metformin use may have some effect on reducing thyroid cancer risk. Metformin is a first-line drug for the treatment of T2DM diabetes and also has a therapeutic effect on obesity and ageing-related diseases. Given the high prevalence of diabetes, the yearly increase in the obese population and the increased risk of having thyroid cancer in patients with diabetes as well as obesity, the effect of metformin on thyroid cancer in our study is promising. However, more well-designed basic and clinical studies are needed to further elaborate these associations.

## Data Availability

The original contributions proposed in the data availability statement study are included in the article and can be further queried to the corresponding author.
